# Agricultural Adaptation to Global Warming in the Tibetan Plateau

**DOI:** 10.3390/ijerph16193686

**Published:** 2019-09-30

**Authors:** Yanling Song, Chunyi Wang, Hans W. Linderholm, Jinfeng Tian, Ying Shi, Jinxia Xu, Yanju Liu

**Affiliations:** 1 State Key Laboratory of Severe Weather, Chinese Academy of Meteorological Sciences, Beijing 100081, China; songyl@cma.gov.cn; 2 Department of Earth Sciences, University of Gothenburg, 405 30 Gothenburg, Sweden; hansl@gvc.gu.se; 3 Department of Geography, University of Cambridge, Cambridge CB2 3EN, UK; 4 Faculty of Agricultural and Nutritional Sciences, Kiel University, 24118 Kiel, Germany; stu115178@mail.uni-kiel.de; 5 National Climate Center, China Meteorological Administration, Beijing 100081, China; shiying@cma.gov.cn (Y.S.); liuyanj@cma.gov.cn (Y.L.); 6 Climate Center of Sichuan Province, China Meteorological Administration, Chengdu 610072, China; xuransige@sina.cn

**Keywords:** global warming, agricultural adaptation, Tibetan plateau

## Abstract

The Tibetan plateau is one of the most sensitive areas in China and has been significantly affected by global warming. From 1961 to 2017, the annual air temperature increased by 0.32 °C/decade over the Tibetan Plateau, which is the highest in the whole of China. Furthermore, this is a trend that is projected to continue by 0.30 °C/decade from 2018 to 2050 due to global warming using the Regional Climate Model version 4 (RegCM4). The increased temperature trend in recent decades has been highest in winter, which has been positive for the safe dormancy of winter wheat. In order to investigate agricultural adaptation to climate change in the Tibetan plateau, we used the World Food Studies (WOFOST) cropping systems model and weather data from the regional climate model RegCM4, to simulate winter wheat production in Guide county between 2018 and 2050. The simulated winter wheat potential yields amounted to 6698.3 kg/ha from 2018 to 2050, which showed the wheat yields would increase by 81%, if winter wheat was planted instead of spring wheat in the Tibetan Plateau with the correct amount of irrigation water. These results indicate that there are not only risks to crop yields from climate change, but also potential benefits. Global warming introduced the possibility to plant winter wheat instead of spring wheat over the Tibetan Plateau. These findings are very important for farmers and government agencies dealing with agricultural adaptation in a warmer climate.

## 1. Introduction

Climate change has attracted much attention in research, as well as from the general public in recent years. A large number of studies focusing on temperature changes have shown that temperature is increasing worldwide [1]. Regional temperature has also increased in China, and the average annual temperature for the whole of China increased by 1.17 °C (0.23 °C/10a) from 1901 to 2016, the strongest regional increase was observed on the Tibetan Plateau (TP) with 0.37 °C/10a, showing high sensitivity to global warming [2]. Like global temperatures, which are projected to increase due to increasing concentrations of atmospheric carbon dioxide and other greenhouse gases [1], the temperature is projected to continue to increase in China [3]. 

Climate change has profoundly influenced agriculture, especially in China. Increasing evidence suggests that there is little doubt that, in association with increasing temperature, the length of the growing season has been extended over the 20th century [4,5,6,7,8,9], meaning crops have more time and better thermal conditions for growth. While the frequency and intensity of meteorological disasters caused by climate change increased, droughts, for example, were more serious due to less precipitation and higher temperatures in China [10], and drought losses in China might double between 1.5 °C and 2.0 °C global warming above the preindustrial at 2020–2039 and 2040–2059 [11]. Meanwhile, cold events for crops have decreased in China [12]. As a result, there are not only risks to crop yields from climate change but also potential benefits. In most of the regions in China, climate change has had a negative impact on crop production [13,14,15,16,17]. While in highly sensitive regions, like the TP, there has been little research on the influence of global warming on yields or production of crops, as well as on the agricultural adaptation to global warming. Further, most farmers in Xizang only have an annual income of about $1300, which is only about 38% of the average resident’s income over the whole of China [18]. So, as most farmers on the TP live in poverty, crop production is a very important source of income.

Spring wheat is grown in the east of the TP, but yields are lower than those of winter wheat. Droughts and low temperatures are the main factors which can influence the growth of wheat. Spring wheat is usually planted in April and harvested in August. In previous decades, winter wheat could not be planted in the TP due to the low temperatures in winter over most of the TP. Winter wheat needs to be planted in autumn (September to October), requires a two to three month vernalization period, where temperatures should be in the range of 5 °C to 0 °C, and is harvested in the next summer. It has been shown that when the average temperature falls below −10 °C, winter wheat does not have a safe dormancy period [19].

So, given the sensitivity of the TP to climate change and the problems with yields of crops as well as the economic problems of farmers, the general aim of this study was to estimate the impact of climate change on agriculture on the TP over the coming 30 years, using the World Food Studies (WOFOST) cropping systems model, and subsequently suggest measures of adaptation.

## 2. Material and Methods

### 2.1. Study Area

**Figure 1:**
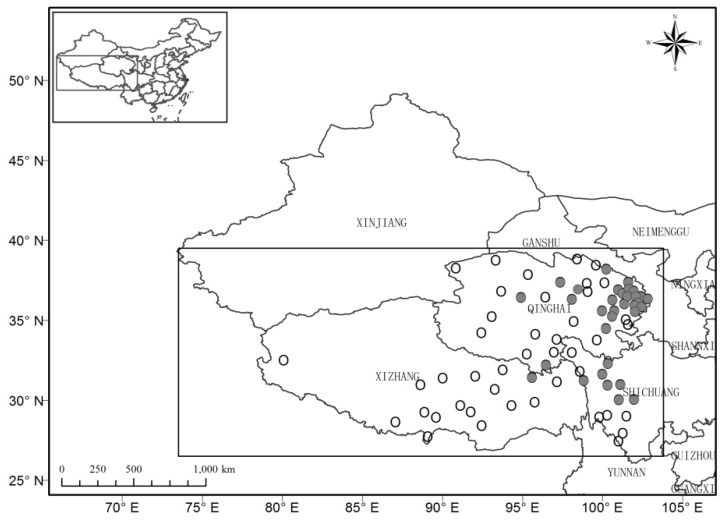
The distribution of weather stations (white circle) and stations in spring wheat regions (grey circles) over the Tibetan plateau (TP) area of China.

The TP, also known as the third pole, is located mainly in the west of China (25° N–40° N and 74° E–104° E), and includes the provinces of Qinghai and Xizang and parts of Sichuan and Yunnan (Figure 1). The TP is the highest plateau in the world, with an altitude ranging from 3000–5000 m, as the west has a higher mean elevation region than the east. The climate of the TP is special due to its high-altitude environment and can be characterized as continental. The annual mean surface air temperature is 3.8 °C (1981 to 2010), which is 9.4 °C lower than the average in China. The total annual precipitation generally ranges from 380–500 mm, being higher in the east. Summers are short, rainy, and warm (monthly mean surface air temperature ranges from 11–14 °C, precipitation ranges from 20–550 mm), and winters are long, cold and very dry (average monthly temperature ranges from −11 to −6 °C, precipitation ranges from 1–40 mm). The climate of the TP dictates the distribution of agriculture and livestock production. The western regions are used mainly for livestock, while crops (wheat, broad beans, and rape) are mainly planted in the east. Triticum aestivum L. is the main staple food crop, with a total farming area of nearly 0.14 million hectares in 2016 in TP [18]. Although the planting area of wheat in TP is small relative to the wheat area of the whole of China, wheat production is very important to local farmers. 

### 2.2. Data 

In order to investigate the agricultural adaptation options to climate change over the TP area, daily precipitation, mean temperatures, and minimum and maximum temperatures for the period 1961–2017 were used, which were provided by the China Meteorological Administration. Only the station series covering periods longer than 57 years with a low rate of missing data (≤5%) were chosen (white circles in Figure 1). In total 66 stations were included in the study.

The characteristics of future climate on the TP were derived from a regional climate model (RCM). Of the various RCMs that have been applied to China, the RegCM series [20,21,22] has been the most commonly used. We used the latest model version: Regional Climate Model version 4 (RegCM4), which is a new version of RegCM developed and updated by The Abdus Salam International Center for Theoretical Physics (ICTP). One representative concentration pathway (RCPs), the most appropriate scenarios for the Paris Agreement’s target of limiting global warming to 2 °C (IPCC, 2013), was considered. RCP4.5 is a scenario of long-term, global emissions of greenhouse gases, and land-use-land-cover which stabilizes radiative forcing at 4.5 Wm^−2^ (approximately 650 ppm CO_2_-equivalent) in the year 2100 [23]. The RegCM4 model projects tendencies of temperature and precipitation and other climate characteristics in China from 2006 to 2050, and the relevant climate parameters were then used to run the World Food Studies (WOFOST) model (see below). 

Particular attention was paid to agricultural adaptation options to global warming, so the yields of spring wheat were applied by the Ministry of Agriculture of the People’s Republic of China. 

### 2.3. Methods

#### 2.3.1. The WOFOST Model

The WOFOST Model was developed by the Wageningen University of Netherland to simulate the effect of cultivar, planting density, weather, soil, water and nitrogen on crop growth, development and yield [24]. The model had been used for crop growth monitoring [25], potential yield forecasting on regional and national scales, as well as for climate change impact scenarios [10,26]. Furthermore, the model had been applied in different climatic regions, including China [26,27,28]. Wu (2003) conducted an experiment for two successive years (2000–2001) at Yucheng Comprehensive Experiment Station in the east of China comparing winter wheat experiments, the simulations showed that the performance of WOFOST was reasonable in China.

The WOFOST model simulates crop growth on the basis of underlying processes, such as photosynthesis and respiration, and how these processes are influenced by environmental conditions. The WOFOST model describes the phenological development, growth, and yield formation, of a crop from emergence to maturity based on crop genetic properties and environmental conditions. The model simulates the dry matter accumulation of a crop as a function of irradiation, temperature, and crop characteristics in one-day time steps. The basis for calculating dry matter production is the rate of gross CO_2_ assimilation of the canopy. This rate depends on the solar radiation absorbed by the canopy and the photosynthetic characteristics of individual leaves from the absorbed radiation. A part of the carbohydrates produced is used to provide for the maintenance of the existing live biomass. The remaining carbohydrates are converted into structural matter.

#### 2.3.2. The Correction of Systematic Errors from RegCM4 

Systematic errors in temperature and precipitation are caused mainly by the climate model simulation as well as by different geographical conditions between the sites and grids in the process of transformations. This bias in turn affects the simulated crop productivity. For example, the growth of a specific crop needs a certain temperature range, and if the simulations provide temperatures that are lower or higher than this range, it will have an adverse effect on the growth of the crop modeled in WOFOST. Therefore, before the application of the projected data, it was necessary to identify and correct any errors between the observed and simulated climates [12].

The mean temperature, maximum temperature, and minimum temperature were corrected using the following method:

(1) $$Correction(cf)=M_{binn}^{RCMscenario}+\left(\bar{M_{binn}^{obs}}-\bar{M_{binn}^{RCMbaseline}}\right)$$

and precipitation was corrected using the following method:

(2) $$Correction(cf)=M_{binn}^{RCMscenario}*\left(\frac{\bar{M_{binn}^{obs}}}{\bar{M_{binn}^{RCMbaseline}}}\right)$$

Correction(cf): The corrected result of the future climate scenario.

$M_{binn}^{RCMscenario}$: The data of the future climate scenario of the climate model simulation in a timescale.

$\bar{M_{binn}^{obs}}$: The average value of historical observations within a timescale.

$\bar{M_{binn}^{RCMbaseline}}$: The average value of historical simulations from a climate model within a timescale.

## 3. Results and Discussion

### 3.1. The Correction of Systematic Errors from RegCM4

**Figure 2:**
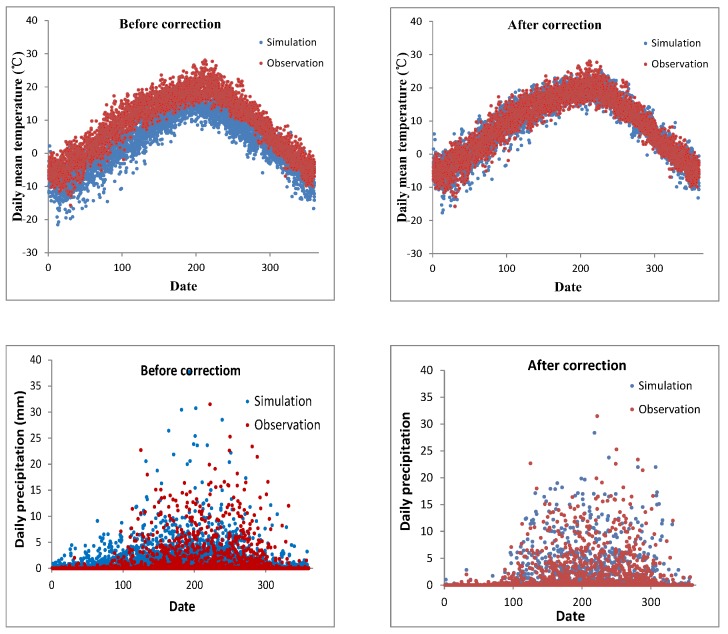
A comparison of observed and RegCM4 simulated daily mean temperatures and precipitation from 2006 to 2015 in Guide station of the Tibetan Plateau, before correction (**left**) and after correction (**right**).

Using the formulae 1 and 2, the temperature and precipitation were corrected in the study area. For example, the correction needed for the simulated temperatures from RegCM4 was analyzed by comparing the observed and simulated temperatures for Guide station. The results showed an obvious gap between simulated and observed temperatures, where the simulated temperatures were generally lower than the observed ones (Figure 2). After the correction, the difference between projected and observed temperatures was smaller than before the correction. Using this method, the projected future climate data was corrected.

### 3.2. Climate Change over the Tibetan Plateau

**Figure 3:**
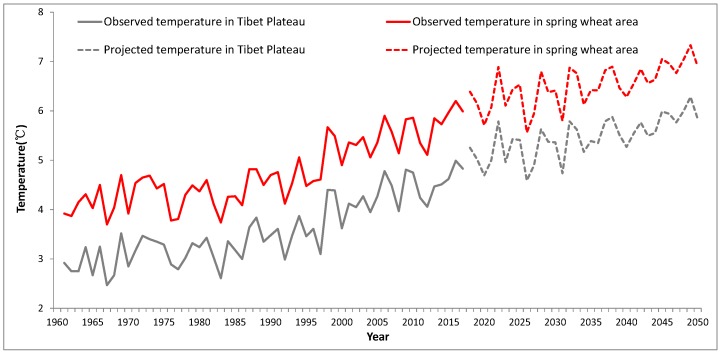

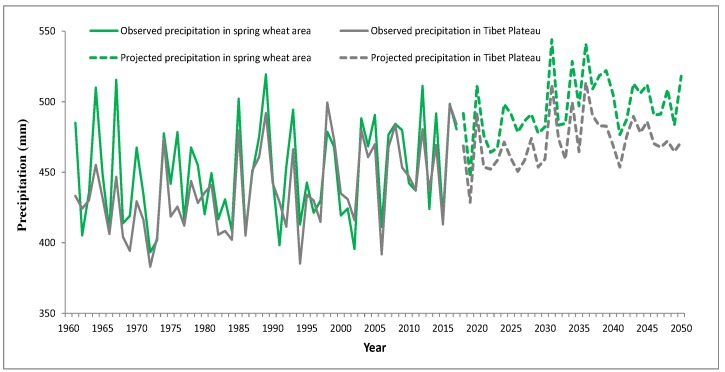
The change of annual temperature and precipitation over wheat area of the Tibetan Plateau of China during the periods 1961–2017 (observed) and 2018–2050 (projected) from REGCM4.

Crops do not in general grow well on the TP due to the low temperatures associated with the high elevation. From 1961 to 2017, the annual mean air temperature increased by 0.32 °C/decade over the TP (Figure 3), which was higher than that of the whole of China. In the TP spring wheat area, the annual mean temperature was about 4.1 °C in the 1960s, and increased to 5.5 °C in the 2000s. It is projected to increase by 0.30 °C/decade from 2018 to 2050, and thus reach an average of 6.9 °C in the 2040s (Figure 3).

Furthermore, the average temperature increased in all seasons, with the increased temperature trend being highest in winter by 0.5 °C/decade from 1961 to 2017. This observed change was favorable for winter wheat, which is not commonly cultivated in the TP due to the low temperatures and low amounts of precipitation during winter. Given the temperature constraints for cultivating winter wheat (see above), increasing winter temperatures could provide the possibility for planting winter wheat on the TP, provided that water is accessible.

**Figure 4:**
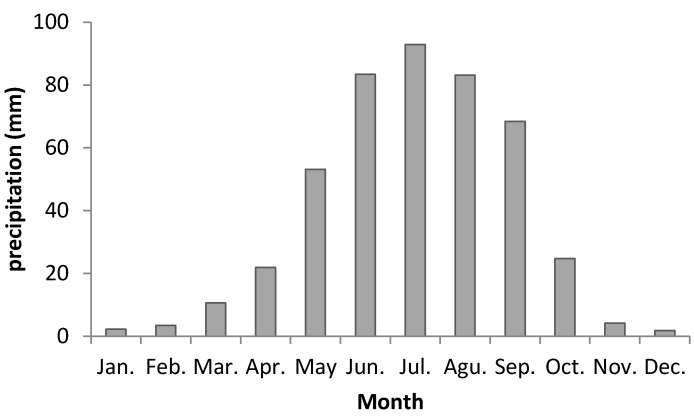
Average monthly precipitation during the period 1981–2010 in the spring wheat regions of the Tibetan plateau area of China.

Meanwhile, the annual precipitation increased by 7.2 mm/decade over the whole of the TP, and 3.7 mm/decade in the spring wheat region over 1961–2017, presently the annual precipitation ranges from 400–500 mm in the spring wheat region. Precipitation is projected to increase slightly by 5.9 mm/decade and 9.0 mm/decade in the whole of the TP and spring wheat region respectively (Figure 3). Spring wheat requires about 350 mm precipitation during its growing season (March to August), and there is 345 mm in a normal year. While the requirement for winter wheat is about 450 mm (October to June), and there is 205 mm in a normal year during the growing season of winter wheat (Figure 4). So, the amount of growing-season precipitation on the TP is sufficient for spring wheat, but is not enough for winter wheat growth, which needs to be irrigated.

### 3.3. Options for Agricultural Adaptation to Climate Change

#### 3.3.1. New Chance of Agriculture in the Tibetan Plateau

In 2010, the cultivated area of spring wheat in the eastern TP amounted to 11.9 × 10^4^ hectare, corresponding to 15.1% of the total planted area in the Qinghai and Xizang provinces [29]. Generally, spring wheat is planted in April, flowers in July, and matures in August. In recent decades (1981–2010), average monthly temperatures/precipitation during the growing season were 6.3 °C/21.9 mm in April, 10.5 °C/53.1 mm in May, 13.6 °C/83.4 mm in June, 15.4 °C/92.9 mm in July, and 14.6 °C/83.1 mm in August, suggesting favorable conditions for spring wheat growth. However, because of the short growing season, spring wheat yields and flour quality are lower than those of winter wheat planted in the North China Plain [29]. While winter wheat should be planted in October, and would be dormant in December, January, and February, it would grow in March again, and then it would flower in June, and be reaped in July. Compared to the five-month growing period of spring wheat, winter wheat needs about 10 months from planting to harvesting, as well as more thermal resource to grow, and produces higher yields and quality. In the past 50 years, spring wheat has been planted in most regions of the TP, but winter wheat could not be planted in most parts of the region because of low winter temperatures.

**Figure 5:**
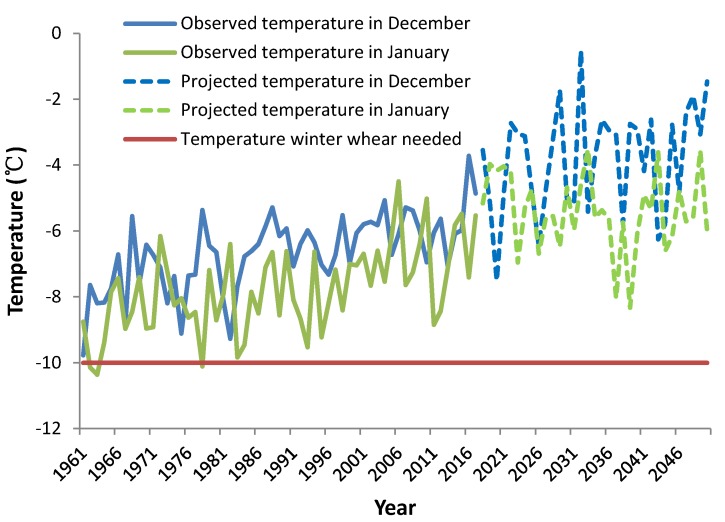
The change of monthly temperatures over the wheat area in December and January during the periods 1961–2017 (observed) and 2018–2050 (projected) from RegCM4.

In Table 1 and Figure 5, the observed and required temperatures for winter wheat are shown, and it is clear that temperatures are sufficient for winter wheat growth in every month except December and January. In other months, winter wheat would grow slowly if temperatures were lower than normal. Although winter wheat is dormant during December and January, the protoplast of wheat root cells will dehydrate and freeze between the cell or between the protoplast and the cell wall if temperatures are lower than −10 °C. The freezing cells increase in volume and are damaged due to the mechanical pressure of the ice crystals. In addition, freezing may cause the cell membrane structure to change, so that the permeability increases and causes electrolyte extravasation, destroying the protoplast and causing the cell to die [29]. Thus, at present, if mid-winter temperatures are lower than normal, there is a considerable risk of harvest failure when planting winter wheat on the TP. Moreover, the average precipitation of 200 mm for the winter wheat growing season (October–July), falls short of the required 400 mm [29]. This can, however, be solved if irrigation is possible. Overall, the temperature in December and January was the controlling factor for winter wheat growth, while the increasing temperature caused by climate change was favorable for growth of winter wheat. The monthly temperature was −7.7 °C in 1961–1970, and −5.7 °C in December in 2008–2017, increasing by 2 °C, and it was −8.8 °C in 1961–1970, and −6.7 °C in January in 2008–2017, increasing by 2.1 °C. Furthermore, the monthly temperature was only −10.1 °C in 1962, −10.4 °C in 1963, −9.4 °C in 1964, which was near or less than −10 °C (Figure 5). The lower temperature could cause winter wheat death, decreasing the yield profoundly. While the monthly temperature increased in 2008–2017, and the lowest monthly temperature was −7.1 °C in December in 2013, and −8.9 °C in January in 2011, which was higher than −10 °C, winter wheat could be dormant safely in 2008–2017. Further, for the future 30 years, the temperature in winter will continue to increase, and the increased temperature in winter will be safe for winter wheat growth.

The yields of spring wheat can amount to 3000–4500 kg/ha, while the yields for winter wheat are more than those of spring yields. This indicates that farmers could produce more wheat if winter wheat was planted instead of spring wheat in some regions over the Tibetan Plateau. As the average annual income of farmers on the TP is only 1300$ [18], increasing the wheat production with winter wheat would increase the incomes of local farmers.

#### 3.3.2. A Case Study of Agricultural Adaptation Options to Climate Change

To study options for agricultural adaptation to climate change on the TP, Guide county (E 101.47°, N 36.02°), which is located in the east of Qinghai province, was chosen. In Guide county, spring wheat is planted over a large area, with 3144.7 ha in 2010. However, in previous decades winter wheat could not be planted due to low winter temperatures. As temperatures have increased due to global warming, there is a potential to grow winter wheat in this region. Furthermore, the Yellow river, which is the second largest river in China, traverses Guide county, providing enough irrigation water to support the growth of winter wheat. Using the WOFOST model, winter wheat yields were simulated using projected climate data, and compared to spring wheat yields. 

The annual temperature is projected to increase by 0.26 °C/decade from 2018 to 2050 using the corrected RegCM4 data, and the annual precipitation to increase by 12.5 mm/decade in Guide county. Using the genetic parameters of winter wheat on the North China Plain [10] and corrected projected weather data from RegCM4, simulations of winter wheat were performed for Guide county for 2018–2050. It was assumed that winter wheat variety and agriculture management practices were consistent through the simulation period, so that the simulated potential yields were only influenced by climate. Moreover, the simulations were made under the assumption that water availability was enough, and consequently the simulated potential yields were mainly affected by temperature. 

**Figure 6:**
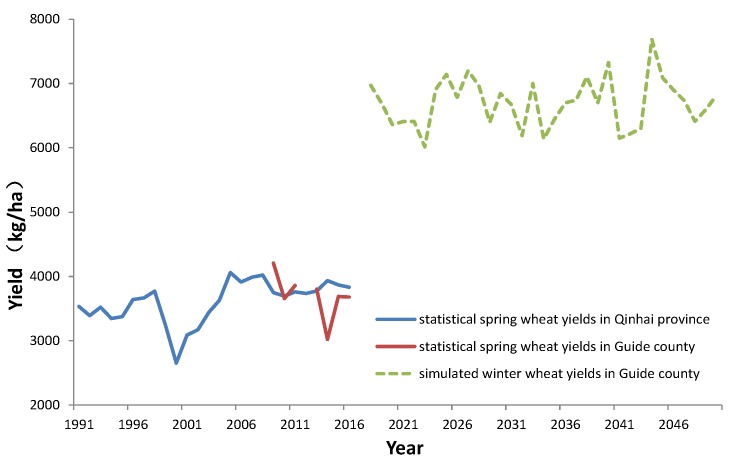
The change of spring wheat yields in the Qinghai province (1991–2016), Guide county (2009–2016), and simulated winter wheat potential yields (2018–2050).

The results showed that the statistical spring wheat yields in Qinghai province was, on average, 3414.4 kg/ha in the 1990s, and 3835.7 kg/ha from 2007–2016 in Guide county. While the simulated winter wheat potential yields amounted to 6698.3 kg/ha from 2018 to 2050. These results indicate that the wheat yields would be increased by 81% if winter wheat was planted instead of spring wheat (Figure 6). The results also indicate the simulated potential yields of winter wheat could fluctuate greatly, as the simulated highest yield of winter wheat was 7690 kg/ha in 2044, while the simulated lowest yield was 6015.4 kg/ha in 2023, which was 21.8% less than in 2044. In a word, the wheat production would be increased substantially if the spring wheat was replaced by winter wheat due to increasing temperature under climate change.

Due to global warming, the annual temperature increased by 0.32 °C/decade in the Tibetan Plateau (Figure 2). Furthermore, the monthly temperature was higher than −10 °C in December and January (Figure 4), which could be positive for winter wheat growth, reducing damage caused by freezing. If winter wheat could replace spring wheat, the simulated potential yields of winter wheat could amount to 6698 kg/ha in Guide county, which is on a similar level to the present yields in North China [10]. The results indicate that the winter wheat yield level of the Tibetan Plateau could reach the yields level of North China if enough irrigation water was available, but the fluctuation of winter wheat yield was larger in the Tibetan Plateau.

## 4. Conclusions

The Tibetan plateau is the highest plateau in the world, with an altitude of 3000–5000 m, so the climate is very special, with low temperature due to the high altitude. Only spring wheat and broad beans could grow well in the low temperature environment with low yield, as a result, most local farmers lived in poverty.

Due to global warming, the region of the Tibetan Plateau became one of the most sensitive areas in China. The annual air temperature increased by 0.32 °C/decade over the Tibetan Plateau from 1961 to 2017, which was a greater increase than anywhere else in China. Furthermore, the average temperature also increased in every season on the Tibetan Plateau, and the increased temperature trend in winter was the highest by 0.5 °C/decade. Also, using RegCM4, the future temperature is projected to increase by 0.30 °C/decade from 2018 to 2050 due to global warming. 

In previous decades, winter wheat could not grow in most regions of the Tibetan Plateau because of low temperatures, especially in winter. Winter wheat would be dormant in December and January. When the monthly temperature was lower than −10 °C, the winter wheat could not be safely dormant and would be damaged or destroyed by frost. Due to global warming, the winter temperature is increasing, with monthly temperatures amounting to −5.7 °C in December and −6.7 °C in January during 2008–2017, which was higher than −10 °C, showing that winter wheat could be safely dormant over the last 10 years. So, there was a possibility to plant winter wheat in the Tibetan Plateau due to global warming. 

In order to investigate the possibility to plant winter wheat in the Tibetan Plateau, using the genetic parameters of winter wheat in China and corrected projected weather data from RegCM4, simulations of winter wheat were performed for Guide county in the Tibetan Plateau from 2018–2050. The results showed that the simulated potential winter wheat yields amounted to 6698.3 kg/ha from 2018 to 2050. These results showed that the wheat yields would be increased by 81% with high fluctuations, if winter wheat was planted instead of spring wheat on the Tibetan Plateau. The results also indicate that the averaged winter wheat yield of the Tibetan Plateau could reach the level of North China if enough irrigation water was provided, but the fluctuation of winter wheat yield would be larger on the Tibetan Plateau. Of course, these simulated higher wheat productions need to be validated with other field trials in TP.

In contrast to spring wheat, winter wheat needs more time and more thermal resources to grow, but winter wheat can produce higher yields and quality. Global warming creates the possibility to plant winter wheat on the Tibetan Plateau, so it is very important for farmers and government to adapt agricultural options to global warming. 

## Figures and Tables

**Table 1:** Mean temperature and precipitation in 1961–1970 and 2008–2017, as well as temperatures needed for winter wheat growth in the wheat regions of the Tibetan Plateau, China.

Month	Winter Wheat Grow Period	T Winter Wheat Needed (°C)	T during 1961–1970	T during 2008–2017
October	Sowing and Seeding	>5	4.7	6.1
November	Seeding and dormancy	>−5	−2.7	−0.59
December	Dormancy	>−10	−7.7	−5.7
January	Dormancy	>−10	−8.8	−6.7
February	Dormancy	>−10	−5.6	−2.9
March	Grow slowly	>0	0.29	1.9
April	Grow fast	>5	6.0	7.1
May	Jointing	>10	10.3	11.1
June	Flowering	>10	12.6	14.3
July	maturity	>14	14.9	16.3
